# A Novel Selective Inhibitor of Delta-5 Desaturase Lowers Insulin Resistance and Reduces Body Weight in Diet-Induced Obese C57BL/6J Mice

**DOI:** 10.1371/journal.pone.0166198

**Published:** 2016-11-10

**Authors:** Hiroaki Yashiro, Shuichi Takagahara, Yumiko Okano Tamura, Ikuo Miyahisa, Junji Matsui, Hideo Suzuki, Shota Ikeda, Masanori Watanabe

**Affiliations:** Pharmaceutical Research Division, Takeda Pharmaceutical Company Limited, Kanagawa Japan; Universidade do Estado do Rio de Janeiro, BRAZIL

## Abstract

Obesity is now recognized as a state of chronic low-grade inflammation and is called as metabolic inflammation. Delta-5 desaturase (D5D) is an enzyme that metabolizes dihomo-γ-linolenic acid (DGLA) to arachidonic acid (AA). Thus, D5D inhibition increases DGLA (precursor to anti-inflammatory eicosanoids) while decreasing AA (precursor to pro-inflammatory eicosanoids), and could result in synergistic improvement in the low-grade inflammatory state. Here, we demonstrate reduced insulin resistance and the anti-obesity effect of a D5D selective inhibitor (compound-326), an orally active small-molecule, in a high-fat diet-induced obese (DIO) mouse model. *In vivo* D5D inhibition was confirmed by determining changes in blood AA/DGLA profiles. In DIO mice, chronic treatment with compound-326 lowered insulin resistance and caused body weight loss without significant impact on cumulative calorie intake. Decreased macrophage infiltration into adipose tissue was expected from mRNA analysis. Increased daily energy expenditure was also observed following administration of compound-326, in line with sustained body weight loss. These data indicate that the novel D5D selective inhibitor, compound-326, will be a new class of drug for the treatment of obese and diabetic patients.

## Introduction

Obesity is generally defined by an excessive fat accumulation, and is recognized as a pandemic nutritional disorder in both developing and developed countries [1–4]. The cause of obesity is usually attributed to a chronic imbalance between energy intake and energy expenditure, and is the cause of complications such as type 2 diabetes (T2DM), dyslipidemia, and cardiovascular disease (CVD). Although individual and genetic factors could influence the onset and severity, the major causative factor for obesity is the excessive intake of fat and carbohydrate, partly related to the western-style-diets spreading across the world [5]. In western-style diets, poly unsaturated fatty acids (PUFA) comprise up to 20% of dietary fat, and linoleic acid (18:2, n-6, LA) and α-linolenic acid (18:3, n-3, ALA) usually contribute more than 95% of dietary PUFA intake, and the diets have low n-3/n-6 PUFA ratio [6–8]. PUFA are essential because they are not synthesized by the body and must be obtained through foods or supplementation [9]. Therefore, dietary intake and food sources of PUFA could influence the whole body PUFA compositions [10].

**Fig 1:**
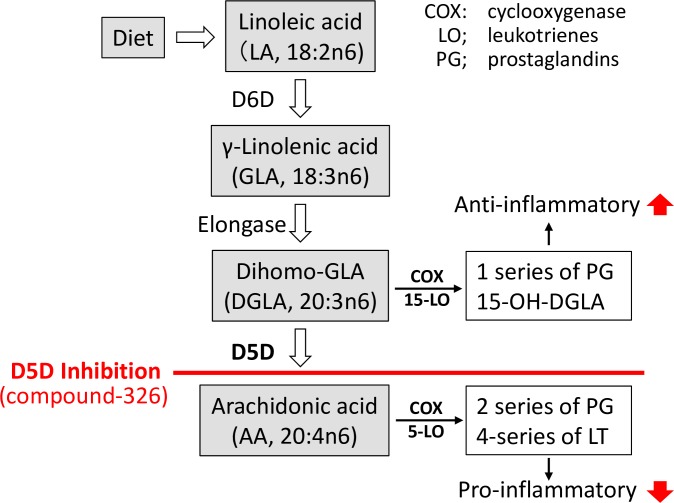
Biosynthesis pathway of n-6 polyunsaturated fatty acids.

Dietary LA is metabolized to dihomo-γ-linolenic acid (20:3, n-6, DGLA) by delta-6 desaturase (D6D; *Fads2*) followed by delta-5 desaturase (D5D; *Fads1*) to arachidonic acid (20:4, n-6, AA), which is a substrate for the synthesis of the pro-inflammatory eicosanoids (2 series of prostaglandins) (Fig 1). An increase in whole desaturase activity, predicted by the enzyme product/enzyme substrate composition, was found in patients with essential hypertension [11], CVD [12], insulin resistance, obesity, and metabolic syndrome [13–15]. Therefore, an increased desaturase activity in combination with an n-6 rich environment may lead to a greater AA accumulation, thus favoring the synthesis of AA-derived mediators, which contribute to chronic inflammation.

It has been reported that the D5D/D6D dual-inhibitor, CP-24879, shows beneficial effects against increased intracellular lipid accumulation and inflammatory injury in hepatocytes [16]. The anti-inflammatory action of this dual inhibitor could mainly be caused by D6D inhibition, which will eventually causes a decrease in AA production. However, D6D inhibition has a more direct impact on reducing DGLA which have anti-inflammatory properties; DGLA is a precursor in the synthesis of prostaglandin E1 (PGE1) as well as 1 series of prostaglandins [17–19]. Therefore, contrary to the D5D/D6D inhibitor, pharmacological inhibition of D5D activity could decrease AA synthesis as well as increase the abundance of DGLA, and synergistically ameliorate metabolic abnormalities by decreasing inflammatory signals, especially in the situation of high LA intake (e.g., Western-style diets).

Meanwhile, D5D is also involved in the metabolic pathway of n-3 PUFA; therefore inhibition of D5D may causes the decrease in *de novo* synthesis of eicosapentaenoic acid (20:5, n-3, EPA) and docosahexaenoic acid (22:6, n-3, DHA) from dietary ALA. Increasing evidence suggests that these n-3 PUFA exert health benefits [20, 21]; thus D5D inhibition may cause some negative impacts on these beneficial effects of n-3 PUFA. On the other hand, recently published paper indicated that D5D knock out (KO) mice showed the phenotype of decreased body fat, improved glucose tolerance, lower fasting serum levels of insulin, cholesterol, and triglycerides compared to wild type mice without abnormal findings [22, 23]; therefore, D5D inhibition could be a therapeutic target for metabolic disease.

Here, we report the discovery of an orally active synthetic small molecule that potently and selectively inhibits D5D, and the therapeutic effect on obesity was evaluated in diet induced obese (DIO) mice. To our knowledge, this is the first report to show the anti-obesity effects of an orally available D5D selective inhibitor in obese animal models.

## Materials and Methods

### Compound

The D5D selective inhibitor, 2-(2,2,3,3,3-Pentafluoropropoxy)-3-[4-(2,2,2-trifluoroethoxy) phenyl]-5,7-dihydro-3H-pyrrolo[2,3-d]pyrimidine-4,6-dione, compound-326 (WO 2010087467A1), was synthesized in Chemical Development Laboratories at Takeda Pharmaceutical Company Limited [24]. Sibutramine hydrochloride monohydrate was purchased from Wako Pure Chemicals (Osaka, Japan). For *in vivo* studies, compounds were suspended in 0.5 w/v% methylcellulose (MC; Wako, Osaka, Japan) solution and administered orally.

### Ethics Statement

The care and use of the animals and the experimental protocols used in this research were approved by the Experimental Animal Care and Use Committee of Takeda Pharmaceutical Company Limited, Japan and the Guide for the Care and Use of Laboratory. During the experimental procedure, we monitored animals every day. Animal conditions were assessed by any symptoms including abnormal behavior, severe anorexia, skin ulceration, and diarrhea. No abnormal findings were noted, and all the mice were well-care and healthy during the experimental procedures. For hepatic-microsomes preparation, rats were sacrificed by decapitation. At the end of all the experiments, mice were sacrificed by exsanguination under pentobarbital anesthesia.

### Animals studies

In this report, we performed 3 independent studies in DIO mice to characterize anti-obesity effects of our D5D specific inhibitor. Male C57BL/6J mice were obtained from Charles River Laboratories (Yokohama, Japan) or CLEA Japan (Osaka, Japan). The mice were fed a laboratory chow and water *ad libitum* and housed in a 12-h light/dark cycle. Compound or vehicle was administered once daily between 15: 00–19:00 by oral gavage. Body weight (BW) and food intake were monitored throughout the experiment.

### *In vitro* enzyme assays for desaturase activities

Delta-9 desaturase (D9D) is a rate-limiting enzyme in the biosynthesis of monounsaturated fatty acids, and it was reported that D9D KO mice demonstrate resistance to diet-induced obesity, increased insulin sensitivity, and increased metabolic rate [25]. Therefore, we also confirmed that compound-326 has no effect on D9D activity. Inhibitory activity of compound-326 on rat D5D, D6D, and D9D was evaluated using hepatic microsomes prepared from SD rats (CLEA, Japan) as described previously [26]. Assays were performed in a total volume of 30 μL in 96-well plates in a buffer containing 100 mM NaHPO_4_, 150 mM KCl, 10 mM NaF, 1.5 mM glutathione, 3 mM MgCl_2_, 1 mM NADH, 3 mM ATP, 0.3 mM CoA-SH, and 0.1% fatty acid free bovine serum albumin (BSA, Sigma, St. Louis, MO); and 0.1 μCi/well of [^14^C] DGLA or [^14^C] linoleic acids (ARC, St. Louis, MO) were used as substrates for measuring D5D and D6D activities, respectively. After pre-incubation of the test compound with rat liver microsomes (final 1.0 mg/mL) for 5 min, the reaction was started by the addition of NADH, ATP, and the substrates, and the reaction mixture was incubated for another 120 min at room temperature. For D9D assay, [^14^C] stearoyl-CoA (PerkinElmer, Waltham, MA) was used in the absence of NADH, ATP, and CoA-SH, and D9D enzyme reaction was performed for 30 min. The reactions were then terminated by the addition of 0.63 N NaOH. After overnight incubation at 55°C for saponification, the solvent extraction of fatty acids was carried out as described previously [27]. The chloroform layer was spotted on a reverse phase TLC plate, RP-18 F254s (Merck Millipore, Darmstadt, Germany), and then developed with acetonitrile: water: acetic acid (95:4.5:0.5). Detection was carried out using BAS-5000 (Fujifilm, Tokyo, Japan). The images were digitalized using Multi Gauge ver. 2.3 (Fujifilm) and IC_50_ values were determined using the conversion ratio from the radioactive substrates (20:3n6, 18:2n6 and 18:0) to the products (20:4n6, 18:3n6 and 18:1n9).

### *In vitro* cell-based assay for desaturase activities

HepG2 cells were seeded in 96-well cell culture plates at 100 000 cells/well and incubated overnight in DMEM supplemented with 10% fetal bovine serum, 100 U/mL penicillin, and 100 μg/mL streptomycin in 5% CO_2_ at 37°C. The cells were washed twice with 200 μL of PBS and incubated with the test compounds in DMEM with 0.1% fatty acid free BSA for 30 min. Then, 0.1 μCi/well of [^14^C]-labeled DGLA, linoleic acid, and stearic acid (ARC, St. Louis, MO) were added for each desaturase assay. After incubation for 3 h at 37°C, the cells were washed 3 times with 200 μL of PBS to remove [^14^C]-labeled substrates in the medium and 0.63 N of NaOH was added to saponify fatty acids. Radioactive substrates and products were extracted, separated, and analyzed using the method as described above.

### Blood and tissue fatty acids measurement

Fatty acids were extracted from blood and tissues by adding hexane-isopropanol-butylated hydroxytoluene (60:40:0.01, v/v/w). Samples were vortexed for 30 min. The hexane phase was removed and evaporated to dryness under nitrogen stream and vacuum. Dried samples were dissolved in methanol and analyzed by HPLC.

### Anti-obesity effects of compound-326 in DIO mice

Mice were fed a high-fat diet (HFD) that has 60% kcal from fat and contains 0.09% (w/w) AA (D12492; Research diets, Inc., see S1 Table for fatty acid profile) from 5 weeks of age, and they were divided into 5 groups (7 animals per group) at 11 weeks of age based on BW and daily food intake. In this experiment, 30 mg/kg sibutramine, a serotonin-dopamine reuptake inhibitor, was used as a positive control for anti-obesity effects. Mice were orally administered compound-326 at doses of 0.1, 1, and 10 mg/kg, sibutramine at a dose of 30 mg/kg, or vehicle for 6 weeks. After 6 weeks, blood concentrations of DGLA and AA under non-fasting condition were evaluated as pharmacodynamics (PD) markers, and oral glucose tolerance test (OGTT) was performed.

### Oral glucose tolerant test

On the last day of the experiment, mice were given an oral glucose load (2 g/kg) after overnight fasting. Blood samples were collected at specified time points of 0 (pre-glucose/fasting glucose levels), 10, 30, 60, and 120 min post glucose load for the determination of plasma glucose as well as insulin levels. Plasma glucose was enzymatically measured with auto-analyzer 7180 (Hitachi High-Technologies Corporation, Tokyo, Japan). Plasma insulin was measured with mouse insulin ELISA kit (Shibayagi, Gunma, Japan). The total area under the glucose curve was determined from time 0 to 120 min (AUC_0–120 min_) after glucose administration. Homeostasis model assessment-insulin ratio (HOMA-IR) was calculated using the formula: [(fasted PG: mg/dL)×(fasted insulin; μU/mL)×26/405] [28].

### The effect of compound-326 on energy expenditure (EE) in DIO mice

To evaluate the effect on daily EE, DIO mice fed a HFD that has 41% kcal from fat and contains no AA (D12079B; Research diets, Inc., see S1 Table for fatty acids composition) for 50 weeks were used. At 55 weeks of age, DIO mice were divided into two weight-matched groups (7 animals per group) and treated with vehicle or 10 mg/kg compound-326 for 11 weeks. The dose of compound-326, 10 mg/kg, was selected that showed clear BW reduction in the previous study. Indirect calorimetry test was performed on day1, 8, 15, 21, and 56 during the study. Daily EE was analyzed using ANCOVA with body mass as covariate [29]. After the treatment period, mice were sacrificed and fat tissues were harvested to evaluate the weights.

### Indirect calorimetry test

Energy metabolism was evaluated using an Oxymax indirect calorimetry system (Columbus Instruments, Columbus, OH). Mice were individually housed in the chamber with a 12-h light/dark cycle. VO_2_ and VCO_2_ rates were determined with the following Oxymax system settings: air flow, 0.6 L/min; sample flow, 0.5 l/min; settling time, 10 min; and measuring time, 1 min. The system was calibrated against a standard gas mixture to measure O_2_ consumed (VO_2,_ mL/kg/h) and CO_2_ generated (VCO_2_, mL/kg/h). Metabolic rate (VO_2_) and respiratory exchange ratio (RER; ratio of VCO_2_/VO_2_) were evaluated over a 24-h period. Energy expenditure was calculated as the product of the calorific value of oxygen (3.815+1.232×RER) and the volume of O_2_ consumed.

### Effects of compound-326 on the expression of inflammation-related genes

Mice fed a HFD that has 41% kcal from fat and contains no AA (D12079B; Research diets, Inc.) from 5 weeks of age were divided into 2 groups at the age of 8 weeks (8 animals per group), and were administered 10 mg/kg compound-326 or vehicle for 6 weeks. After the treatment period, mice were sacrificed and epididymal and subcutaneous fats were harvested for mRNA analysis. Nine animals maintained on a standard chow (CE2, CLEA Japan, Osaka) were used as control.

### RNA extraction and quantitative real-time polymerase chain reaction (RT-qPCR)

Total RNA was extracted and purified using RNeasy Mini Kit (QIAGEN, USA). First-strand cDNA was synthesized using High Capacity cDNA Reverse Transcription Kits (Thermo Fisher, USA). The mRNA levels were quantified by an ABI Prism 7900 TaqMan PCR system (Thermo Fisher, USA) according to the manufacturer’s instruction using TaqMan® Universal PCR Mastermix with the primer-probe sets of TaqMan Gene Expression Assays (Thermo Fisher, USA) for the following genes: *Ccl2* (chemokine (C-C motif) ligand 2; Mm99999056_m1), *Cd68* (CD68 antigen; Mm03047343_m1), *Adgre1* (adhesion G protein-coupled receptor E1; Mm00802529_m1), *Tnf* (tumor necrosis factor; Mm00443258_m1), *Il6* (interleukin 6; Mm99999064), *Lep* (leptin; Mm00434759_m1), *Adipoq* (adiponectin; Mm00456425_m1), *Fads2* (D5D; Mm00517221_m1), *Fads1* (D6D; Mm00507605_m1), *Ucp1* (uncoupling protein 1; Mm00494069_m1), *Elovl3* (elongation of very long chain fatty acids 3; Mm00468164_m1), *Cidea* (cell death-inducing DNA fragmentation factor, alpha subunit-like effector A; Mm00432554_m1), *Otop1* (otopetrin 1; Mm00554705_m1), and *Rplp0* (ribosomal protein, large, P0; forward; 5'-CCCTGAAGTGCTCGACATCAC-3', reverse; 5'-GCGCTTGTACCCATTGATGA-3', probe (VIC-5'→3'-TAMRA); 5'-CCCTGCACTCTCGCTTTCTGGAG-3'). The *Rplp0* mRNA level was used as a reference and relative transcript levels were calculated with the comparative Ct method (2^-ΔΔCT^) (Applied Bio-systems).

### Statistics

Results are expressed as mean ± *SE*. Statistical analysis between two groups was assessed by Aspin-Welch test or Student’s t-test depending on equal or unequal variances. For these analyses, a *p*-value less than 0.05 was considered significant. The dose-dependent effect of compound-326 vs. vehicle was evaluated by the one-tailed Williams’ test or Shirley-Williams test depending on equal or unequal variances, and a *p*-value less than 0.025 was considered significant.

## Results

### Effect of compound-326 on PUFA-desaturases *in vitro*

**Fig 2:**
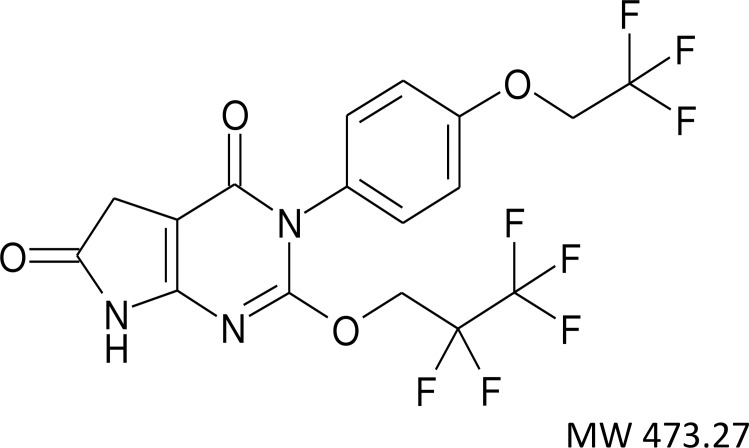
Structure of compound-326.

To obtain useful compounds for exploring D5D selective inhibition, we focused our early efforts on the discovery of a potent, selective, and orally bioavailable D5D inhibitor. We identified compound-326 (Fig 2) [24] which satisfied all these conditions, and its therapeutic potential was evaluated. As shown in Table 1, compound-326 has a potential to inhibit D5D activity (IC_50_ = 72 and 22 nM for rat and human D5D, respectively); whereas, almost no inhibition was observed on both rat and human D6D activities. We also confirmed that compound-326 has no effect on D9D activity.

**Table 1:** *In vitro* IC_50_ values (nM) for fatty acid desaturases.

Compound Name	Assay Methods	Species	D5D	D6D	D9D
Compound-326	Enzyme	Rat	72	>10 000	>10 000
	(hepatic microsomes)				
	Cell-based	Human	22	>10 000	>10 000
	(HepG2)				

### Anti-obesity effects of compound-326 in DIO mice

**Fig 3:**
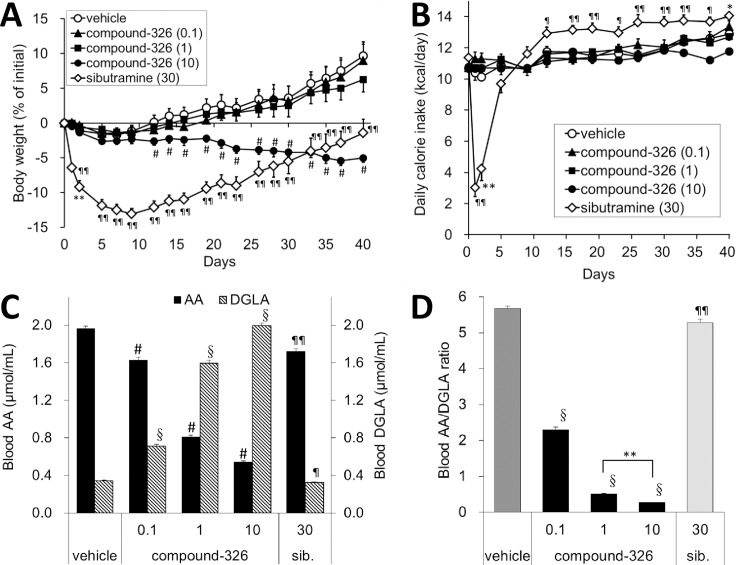
Effects of chronic treatment with compound-326 on BW, calorie intake, and blood PD markers in DIO mice. DIO mice (initial BW; 31.1±1.6 g) were treated with compound-326 (0.1, 1, and 10 mg/kg), 30 mg/kg sibutramine, or vehicle p.o. for 6 weeks. (**A**) Changes in BW from initial value. (**B**) Daily calorie intake during the study. (**C**) Changes in blood AA and DGLA concentrations after 6-week treatment. (**D**) Changes in the blood AA to DGLA ratio after 6-week treatment. Data are expressed as mean ± *SE* (n = 7). #*p*≤ 0.025 vs. DIO vehicle by Williams' test. §*p*≤ 0.025 vs. DIO vehicle by Shirley-Williams test. *p≤ 0.05, **p≤ 0.01 vs. DIO vehicle by Aspin-Welch test. ¶*p*≤ 0.05, ¶¶*p*≤ 0.01 vs. DIO vehicle by Student's t-test.

**Fig 4:**
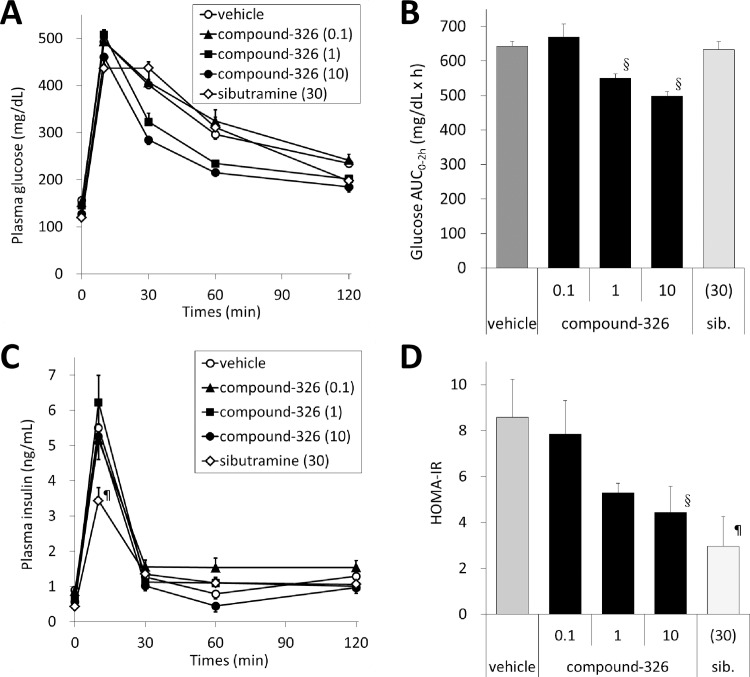
Effects of chronic treatment with compound-326 on plasma glucose and insulin during an OGTT, and HOMA-IR in DIO mice. Oral glucose tolerance test (OGTT) was performed after chronic treatment with compound-326 for 6 weeks in DIO mice. (**A**) Changes in plasma glucose levels. (**B**) Effect on plasma glucose AUC. (**C**) Changes in plasma insulin levels. (**D**) Effect on HOMA-IR. Data are expressed as mean ± *SE* (n = 7). §*p*≤ 0.025 vs. DIO vehicle by Shirley-Williams test. ¶*p*≤ 0.05 vs. DIO vehicle by Student's t-test.

To examine the anti-obesity potential of compound-326, we administered compound-326 at doses of 0.1, 1, and 10 mg/kg orally to DIO mice for 6 weeks. As shown in Fig 3A, mice treated with vehicle only showed increased BW during the experiment, and the sibutramine group showed transient and potent decrease in food intake from day 1, followed by a rebound. The sibutramine group showed potent BW loss for a week, but BW increased in line with increased food intake. On the other hand, the group treated with 10 mg/kg of compound-326 showed gradual decrease in BW; however, lower doses were not effective. Unlike sibutramine, daily calorie intake was not altered significantly due to compound-326 administration at all doses; although slight decrease was observed at a dose of 10 mg/kg (Fig 3B). No abnormal findings such as behavioral changes, diarrhea, or steatorrhea were observed by compound-326 treatment. To confirm D5D inhibition by compound-326 *in vivo*, we determined blood AA and DGLA levels after 6 weeks of treatment. Compound-326 decreased blood AA in conjunction with increased blood DGLA, in a dose-dependent manner (Fig 3C); therefore, the AA to DGLA ratio decreased significantly (Fig 3D). Since there was a large difference in BW loss between 1 and 10 mg/kg of compound-326, we analyzed statistical difference in the blood AA/DGLA profiles between these two doses. Although the changes were small, statistical difference was observed between the doses. Sibutramine treatment slightly but significantly decreased both blood AA and DGLA levels as well as the AA to DGLA ratio, which could be correlated to the reduction in BW. Because D5D is also active in the metabolic pathway of n-3 PUFA, we evaluated the effects of compound-326 on blood levels of EPA and DHA. As shown in S1 Fig, both n-3 PUFA were decreased by compound-326 in a dose dependent manner. An OGTT was performed after 6-week treatment, and glucose clearance was found to be improved by compound-326 in a dose-dependent manner (Fig 4A and 4B), while no change was observed in the insulin levels (Fig 4C). In line with improved glucose clearance, HOMA-IR, an insulin resistance index, was also decreased by compound-326 in a dose-dependent manner (Fig 4D). Treatment with sibutramine significantly improved HOMA-IR, but glucose clearance, as measured by the OGTT, was not improved.

### The effect of compound-326 on daily EE in DIO mice

**Fig 5:**
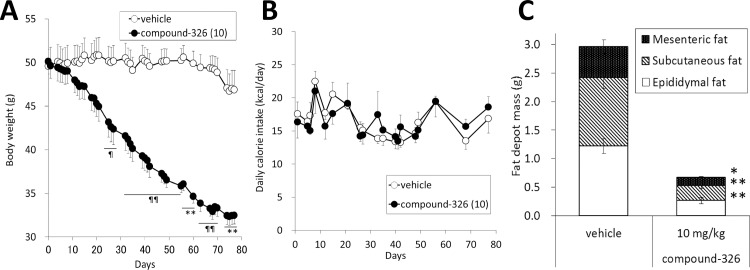
Effects of chronic treatment with compound-326 on BW, calorie intake, and fat mass in DIO mice. DIO mice were treated with 10 mg/kg compound-326, p.o., for 11 weeks. (**A**) Changes in BW. (**B**) Changes in daily calorie intake. (**C**) Weights of different body fat depots after 11-week treatment. Data are expressed as mean ± *SE* (n = 7). *p≤ 0.05, **p≤ 0.01 vs. DIO vehicle by Aspin-Welch test. ¶*p*≤ 0.05, ¶¶*p*≤ 0.01 vs. DIO vehicle by Student's t-test.

**Fig 6:**
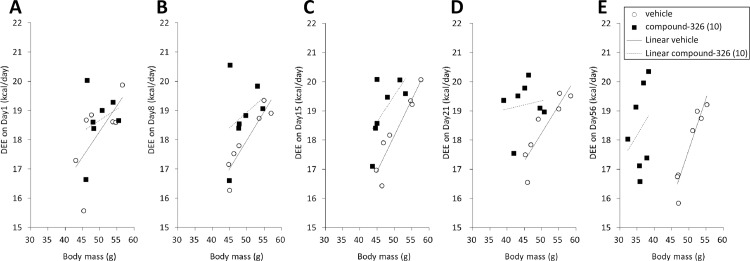
Effect of chronic treatment with compound-326 on energy expenditure in DIO mice. Daily energy expenditure (DEE) was monitored on days 1 (**A**), 8 (**B**), 15 (**C**), 21 (**D**), and 56 (**E**) during the study indicated in Fig 5.

As indicated above, compound-326 prevented obesity and ameliorated insulin resistance without any appreciable decrease in calorie intake. Hence, we next confirmed the amelioration of obesity by compound-326 using established DIO mice that had been fed a HFD for 50 weeks before the experiment. There was no significant difference between the groups in BW prior to treatment (vehicle; 50.4 ± 5.1 g, 10 mg/kg compound-326; 50.9 ± 3.8 g). Consistent with the results obtained in the HFD-fed growing DIO model, chronic treatment with compound-326 caused sustained and significant BW reduction from day 21 until day 77 (Fig 5A). Intriguingly, while no appreciable change in cumulative calorie intake was observed upon compound-326 treatment (data not shown), daily calorie intake was slightly decreased until day 21, followed by a trend toward increase (Fig 5B). Mice treated with compound-326 for 11 weeks showed marked decrease in the white adipose tissue (WAT) depot mass compared to vehicle-treated mice; mesenteric fat (*p*≤ 0.05), subcutaneous and epididymal fat (*p*≤ 0.01) (Fig 5C). Since observed loss in calorie intake could not explain remarkable weight loss, changes in EE were monitored on days 1, 8, 15, 21, and 56, and daily variations are shown in S2 Fig. To evaluate the effect on EE, general linear model analysis was applied on each point. On days 1 and 8, no difference was observed between vehicle- and compound-326-treated groups when daily EE (kcal/day) was plotted against body mass (Fig 6A and 6B). However, on day 15, daily EE lay on two separate lines relative to body mass (Fig 6C), and these changes demonstrate that EE was increased by compound-326 treatment independent of the decreased BW [30]. Moreover, the difference between these lines became more apparent on days 21 and 56, consistent with the amelioration of obesity (Fig 6D and 6E). Compound-326-treated mice showed lower RER relative to vehicle-treated mice through the study, but the change did not reach significance (S3 Fig).

### Effects of compound-326 on gene expression in fat tissues

**Fig 7:**
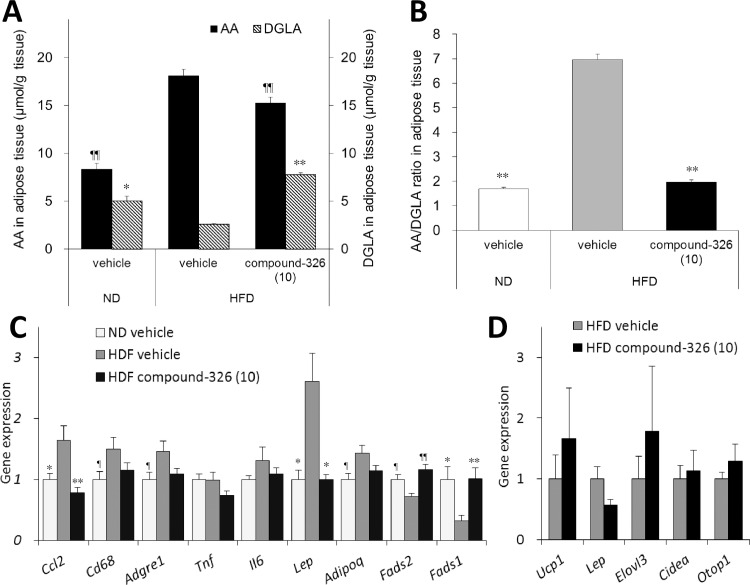
Effects of chronic treatment with compound-326 on PD markers and gene expression in WAT in DIO mice. HFD-fed DIO mice were treated with compound-326 (10 mg/kg), p.o. for 6 weeks. Mice fed a normal diet (ND) were used as normal reference. (**A**) Levels of AA and DGLA in epididymal adipose tissue. (**B**) AA to DGLA ratio in epididymal adipose tissue. RT-qPCR was performed in epididymal (**C**) and subcutaneous fat tissues (**D**) after 6-week treatment. Data are expressed as mean ± *SE* (n = 8–9). *p≤ 0.05, **p≤ 0.01 vs. DIO vehicle by Aspin-Welch test. ¶*p*≤ 0.05, ¶¶*p*≤ 0.01 vs. DIO vehicle by Student's t-test.

To further characterize the effects of our D5D inhibitor, we evaluated changes in the expression of genes related to inflammation and metabolic processes in fat tissues. DIO mice treated with 10 mg/kg compound-326 showed a resistance to obesity, and 6-week treatment significantly decreased BW compared to vehicle treatment (S4A and S4B Fig). In addition, a decrease in the AA to DGLA ratio in the blood, as a consequence of decreased AA and increased DGLA, was confirmed in the compound-326-treated group (S4C and S4D Fig). We also evaluated AA and DGLA contents in the liver (S4E and S4F Fig) as well as in epididymal fat tissue (Fig 7A and 7B). In HFD-fed DIO mice, the AA to DGLA ratio in the liver was significantly decreased compared to ND-fed mice; HFD-feeding increased both DGLA and AA, and the increase in DGLA overcame the increase in AA in the liver (S4E and S4F Fig). The pattern of AA and DGLA changes in the liver are the same as in the blood (S4C and S4D Fig). Conversely, the AA to DGLA ratio in epididymal fat was significantly increased in HFD-fed DIO mice compared to ND-fed mice; DGLA was significantly decreased by HFD-feeding, while AA was increased in epididymal fat tissue (Fig 7A and 7B). These results indicate that changes in the AA to DGLA ratio, namely the D5D activity, in metabolically unhealthy conditions could be differ in tissues, and its changes in the blood seem to reflect the liver conditions. However, compound-326 significantly decreased the AA to DGLA ratio in both tissues, and the change in AA and DGLA in the blood could be a reliable PD marker to confirm the action of the D5D inhibitor in tissues. We also evaluated the effect of compound-326 on EPA and DHA levels in blood and tissues (S5 Fig); however, liver EPA level in compound-326-treated DIO mice and DHA level in adipose tissue of DIO mice were not measureable due to the analytical detection limit. ND-fed mice showed much higher levels of EPA and DHA compared to HFD-fed DIO mice possibly due to the difference in the PUFA composition of the meal; CE2 diet contains fish-meal, while HFD do not contain any EPA or DHA (S1 Table 1). As expected, compoud-326 significantly decreased both n-3 PUFA in blood, liver, and adipose tissue.

In epididymal fat, the expression levels of *Ccl2*, *Cd68*, and *Adgre1* were significantly higher in HFD-fed DIO mice compared to these in ND-fed mice (Fig 7C). And these genes were decreased by chronic treatment with compound-326; while significant effect of compound-326 was only observed in *Ccl2*. As for *Tnf* and *Il6* genes, up-regulation of these genes were not clearly observed in our HFD-fed DIO model; *p* value of *Tnf* and *Il6* between HFD-fed DIO and ND-fed mice are 1.00 and 0.46, respectively. Effects of compound-326 on these inflammation-related genes in epididymal fat were also evaluated using satellite DIO mice that had been treated with compound-A or vehicle for 32 days (S6 Fig). At the intermediate point, compound-326 significantly decreased gene expression levels of *Ccl2*, *Cd68*, *Adgre1*, and *Il6* (S6A Fig), while no significant BW loss was observed (S6B Fig). Compound-326 treatment also decreased epididymal *Lep* and *Adipoq* mRNAs which were significantly increased in HFD (Fig 7D); the effects did not reach statistical difference in *Adipoq* mRNA (p = 0.08). HFD-fed DIO mice showed significantly lower D5D and D6D mRNA expression in epididymal fat, and compound-326 treatment upregulated their expression. In subcutaneous fat, no induction of beige fat cell-selective genes, including *Ucp1*, *Cidea*, *Elovl3*, and *Otop1* were detected by compound-326 treatment (Fig 7D). *Lep* mRNA in subcutaneous fat was decrease by compound-326, but the difference did not reach statistical significance (*p* = 0.08).

## Discussion

Considering the physiological importance of PUFA, D5D and D6D activities likely to regulate a number of metabolic and inflammatory pathways. In addition, the use of product-to-precursor ratio as surrogate to estimate desaturase activity is well stablished, and this method has been used in epidemiological studies [31]. In such studies, D6D has received much more attention compared to D5D, with respect to metabolic disorders, because data demonstrated strong positive correlations between increased D6D activity and insulin resistance, BMI, and T2DM, while D5D activities showed negative correlation [15, 32–36]. Additionally, metabolic benefits of D6D inhibition were also predicted from the results that D6D KO mice exhibited resistance against HFD-induced obesity [37].

Regarding D5D, Powell *et al*. recently reported that D5D KO mice showed lean phenotype with improved glycemic control and decreased development of atheromatous plaque under HFD-feeding [23]. Hence, although there is no clear human evidence available to support the metabolic benefits of D5D inhibition, the concept is similar to D6D inhibition; it will block the supply of AA, which is required for the production of pro-inflammatory eicosanoids. In addition, D5D inhibition increases the abundance of DGLA, which is a known precursor for anti-inflammatory eicosanoids. Thus, pharmacological inhibition of D5D activity may synergistically ameliorate metabolic abnormalities by decreasing inflammatory signals due to the decrease in AA/DGLA ratio.

In the first study, we found that 6-week treatment with compound-326 prevented HFD-induced obesity with a slight decrease in daily calorie intake; although it was not enough to explain the remarkable change in BW. Here, we used sibutramine as a positive control for anti-obesity effect, which produces anorexigenic effects by norepinephrine, serotonin, and dopamine reuptake inhibition. Intriguingly, although both sibutramine and compound-326 showed potent loss in BW, effects on food intake were completely different.

As indicated above, fatty acid product-to-precursor ratio in the blood has been widely used to estimate PUFA desaturase activity [31]; hence, we used changes in the blood AA to DGLA ratio to estimate the inhibition of D5D activity by the test compound. As expected from our *in vitro* studies, blood samples from compound-326-treated mice showed decreased AA and increased DGLA in a dose-dependent manner, and these results indicate that compound-326 has a potential to inhibit D5D activity *in vivo*. Although a significant change in BW could only be observed at a higher dose (10 mg/kg) having a high impact on blood AA and DGLA levels, improvement in glucose tolerability and insulin sensitivity were observed from dose of 1 mg/kg without a significant decrease in BW. These results suggest that near complete inhibition of D5D may be require for loss of BW, while partial inhibition may be enough to improve insulin sensitivity.

We also evaluated anti-obesity effect of compound-326 in established DIO mice, and chronic treatment with 10 mg/kg compound-326 reduced BW, but had a lower effect on calorie intake. While short-term treatment with compound-326 did not increase EE, chronic administration increased EE in line with BW reduction. These results suggest that increased EE by compound-326 may require modified cellular AA and DGLA profiles, which would require a longer period of time.

Although compound-326 treatment could exert an influence on AA and DGLA profiles in the whole body, here we focused on the anti-inflammatory response in adipose tissue, because inflammation originating from excessive amount of visceral fat is considered one of the main driving forces in the development of metabolic abnormality in obese individuals. CCL2, a potent chemotactic factor for monocyte, is produced predominantly by macrophages and its abundance in adipose tissue is increased in obese mice [38]. In the adipose tissue, increased CCL2 signaling through its receptor, CCR2, links obesity and insulin resistance through the induction of an inflammatory response [39]. We found that macrophage-related genes, exemplified by *Ccl2*, *Adgre1*, and *Cd68* were upregulated in epididymal fat of HFD-fed DIO mice compared to normal mice, and chronic treatment with compound-326 decreased the expression of these genes, thereby improving inflammation could be expected in the adipose tissue while further studies are necessary in order to clarify the association. We also confirmed a decrease in the AA to DGLA ratio in epididymal fat due to the administration of compound-326, and these changes could be contribute toward improved insulin sensitivity by suppressing systemic inflammation in the adipose tissue. It is reported that insulin stimulates D5D and D6D gene expression in hepatocytes via a cAMP dependent mechanism [40]. Hence, increased D5D and D6D mRNA expression observed in fat tissue would be caused by improved insulin sensitivity. Leptin is a hormone synthesized and released by WAT, and plays an important role in maintaining energy balance and regulation of BW [41]. In our results, chronic administration of compound-326 led to a marked decrease in leptin mRNA expression in epididymal fat, which could largely be attributed to body fat loss and improved leptin resistance.

Body fat accumulation, and consequently increase in BW, fluctuates in accordance with the difference between energy intake and energy expenditure over time. Here, we examined energy balance in detail and found that chronic administration of compound-326 significantly increased daily EE. The increase in EE with a concomitant slight decrease in energy intake resulted in a severe negative energy balance responsible for the observed fat and weight loss. Some interventions, which modulate metabolic fluxes in adipocytes, result in a general decrease in the accumulation of body fat. Pharmacological stimulation of PPARα [42] or leptin treatment [43] increase UCP1 positive cells in WAT, which mediate thermogenesis and mitochondrial uncoupling, and they are known as beige fat cells. It was reported that dietary AA has a negative influence on the production of beige fat cells upon β3-adrenergic receptor stimulation [44]. Therefore, we evaluated the expression levels of beige fat cell-selective genes such as *Ucp1*, *Elovl3*, *Cid*ea, and *Otop*1 in subcutaneous fat, but no change was observed upon compound-326 treatment in animals.

AA is also the substrate for the synthesis of anandamide and other endocannabinoids. It is well known that rimonabant, a selective inverse agonist of cannabinoid receptor 1 (CB1), has shown to cause weight loss due to decreased food intake and increased energy expenditure [45]. Studies in rodents indicated that chronic rimonabant treatment upregulated UCP1 expression both in interscapular brown adipose tissue (BAT) and WAT adipocytes via increased sympathetic activity [46, 47]. Although compound-326 showed much less impact on food reduction and UCP1 induction in WAT, decreased cannabinoid signaling in BAT may exert increased energy expenditure through a similar mechanism as chronic rimonabant treatment. Thus, while increased energy expenditure by chronic administration of compound-326 may contribute to its anti-obesity effect, further studies are required to delineate the mechanism of the increase in energy expenditure.

Contrary to these beneficial effects of compound-326, there are two major concerns for future development. One of the considerable concerns is the fact that D5D inhibition also affects the n-3 PUFA desaturation pathway. Here, we observed decreased blood and tissue levels of DHA and EPA in compound-326-treated mice. Although any abnormal findings were not observed in the mice treated with compound-326 even in the absence of EPA and DHA intake (S1 Table), this on-target effect may have some negative impact from the therapeutic point of view due to the increasing number of the positive evidence of n-3 PUFA on human health [20, 21]. Another possible concern is the risk of varying efficacy under the different PUFA composition in foods. In the current experiments, we used the HFD that contains high amount of LA and low or no amount of AA (S1 Table), and exemplified the possibility that our D5D inhibitor may exert both anti-inflammatory and anti-obesity effects when taking the high-LA diet, such as western-style diets. On the other hand, dietary intake of AA or other PUFA may attenuate the therapeutic efficacy of our D5D inhibitor in line with the increase in blood and tissue AA/DGLA ratio. Therefore, more experiments are necessary to elucidate the mechanism of action of compound-326 on anti-obesity and anti-inflammatory potentials, and clear mitigation plans for each potential concerns/problems are required.

In conclusion, we developed a D5D selective inhibitor, and showed the availability of the blood AA to DGLA ratio as a useful PD marker. We also confirmed a sub-chronic effect of the novel D5D selective inhibitor, compound-326, on insulin sensitivity and body fat loss with increased energy expenditure. Although further characterization of this D5D selective inhibitor is required to understand the physiological and pathophysiological impact on PUFA-desaturase pathways, this D5D selective inhibitor will be a new class of drug for the treatment of obese and diabetic patients.

## Supporting Information

S1 Fig Effects of chronic treatment with compound-326 on blood EPA and DHA levels in DIO mice. (DOCX)

S2 Fig Effect of chronic treatment with compound-326 on energy expenditure in DIO mice. (DOCX)

S3 Fig Effect of chronic treatment with compound-326 on respiratory exchange ratio in DIO mice. (DOCX)

S4 Fig Effects of compound-326 on BW and AA and DGLA levels within the blood as well as liver in DIO mice. (DOCX)

S5 Fig Effects of chronic treatment with compound-326 on blood and tissue EPA and DHA levels in HFD-fed mice. (DOCX)

S6 Fig Effects of compound-326 on WAT gene expression in satellite DIO mice (Day32). (DOCX)

S1 Table Fatty acids composition of the high-fat diet (data are expressed as w/w%). (DOCX)

## References

[1] Mendis S, Lindholm LH, Mancia G, Whitworth J, Alderman M, Lim S, et al World Health Organization (WHO) and International Society of Hypertension (ISH) risk prediction charts: assessment of cardiovascular risk for prevention and control of cardiovascular disease in low and middle-income countries. J Hypertens. 2007; 25: 1578–1582. 10.1097/HJH.0b013e3282861fd3 17620952

[2] Chopra M, Galbraith S, Darnton-Hill I. A global response to a global problem: the epidemic of overnutrition. Bull World Health Organ. 2002; 80: 952–958. 12571723 PMC2567699

[3] Monteiro CA, Moura EC, Conde WL, Popkin BM. Socioeconomic status and obesity in adult populations of developing countries: a review. Bull World Health Organ. 2004; 82: 940–946. doi: /S0042-96862004001200011 15654409 PMC2623095

[4] Haidar YM, Cosman BC. Obesity epidemiology. Clin Colon Rectal Surg. 2011; 24: 205–210. 10.1055/s-0031-1295684 23204935 PMC3311487

[5] Pan A, Malik VS, Hu FB. Exporting diabetes mellitus to Asia: the impact of Western-style fast food. Circulation. 2012; 126: 163–165. 10.1161/CIRCULATIONAHA.112.115923 22753305 PMC3401093

[6] Simopoulos AP. The importance of the ratio of omega-6/omega-3 essential fatty acids. Biomed Pharmacother. 2002; 56: 365–379. 12442909 10.1016/s0753-3322(02)00253-6

[7] Simopoulos AP. Importance of the ratio of omega-6/omega-3 essential fatty acids: evolutionary aspects. World Rev Nutr Diet. 2003; 92: 1–22. 14579680 10.1159/000073788

[8] Glaser C, Heinrich J, Koletzko B. Role of FADS1 and FADS2 polymorphisms in polyunsaturated fatty acid metabolism. Metabolism. 2010; 59: 993–999. 10.1016/j.metabol.2009.10.022 20045144

[9] Covington MB. Omega-3 fatty acids. Am Fam Physician. 2004; 70: 133–140. 15259529

[10] Astorg P, Bertrais S, Laporte F, Arnault N, Estaquio C, Galan P, et al Plasma n-6 and n-3 polyunsaturated fatty acids as biomarkers of their dietary intakes: a cross-sectional study within a cohort of middle-aged French men and women. Eur J Clin Nutr. 2008; 62: 1155–1161. 10.1038/sj.ejcn.1602836 17622261

[11] Russo C, Olivieri O, Girelli D, Guarini P, Pasqualini R, Azzini M, et al Increased membrane ratios of metabolite to precursor fatty acid in essential hypertension. Hypertension. 1997; 29: 1058–1063. 9095099 10.1161/01.hyp.29.4.1058

[12] Domei T, Yokoi H, Kuramitsu S, Soga Y, Arita T, Ando K, et al Ratio of serum n-3 to n-6 polyunsaturated fatty acids and the incidence of major adverse cardiac events in patients undergoing percutaneous coronary intervention. Circ J. 2012; 76: 423–429. 22156311 10.1253/circj.cj-11-0941

[13] Inoue K, Kishida K, Hirata A, Funahashi T, Shimomura I. Low serum eicosapentaenoic acid / arachidonic acid ratio in male subjects with visceral obesity. Nutr Metab (Lond). 2013; 10: 25. 23497138 10.1186/1743-7075-10-25 PMC3606329

[14] Vessby B. Dietary fat, fatty acid composition in plasma and the metabolic syndrome. Curr Opin Lipidol. 2003; 14: 15–19. 10.1097/01.mol.0000052859.26236.5f 12544656

[15] Warensjo E, Ohrvall M, Vessby B. Fatty acid composition and estimated desaturase activities are associated with obesity and lifestyle variables in men and women. Nutr Metab Cardiovasc Dis. 2006; 16: 128–136. 10.1016/j.numecd.2005.06.001 16487913

[16] Lopez-Vicario C, Gonzalez-Periz A, Rius B, Moran-Salvador E, Garcia-Alonso V, Lozano JJ, et al Molecular interplay between Delta5/Delta6 desaturases and long-chain fatty acids in the pathogenesis of non-alcoholic steatohepatitis. Gut. 2014; 63: 344–355. 10.1136/gutjnl-2012-303179 23492103

[17] Knapp HR, Oelz O, Whorton AR, Oates JA. Effects of feeding ethyl-dihomo-gamma-linolenate on rabbit renomedullary lipid composition and prostaglandin production in vitro. Lipids. 1978; 13: 804–808. 713717 10.1007/BF02533480

[18] Kirtland SJ. Prostaglandin E1: a review. Prostaglandins Leukot Essent Fatty Acids. 1988; 32: 165–174. 2901111 10.1016/0952-3278(88)90168-8

[19] Zurier RB. Role of prostaglandins E in inflammation and immune responses. Adv Prostaglandin Thromboxane Leukot Res. 1991; 21B: 947–953. 1825439

[20] Dyall SC. Long-chain omega-3 fatty acids and the brain: a review of the independent and shared effects of EPA, DPA and DHA. Front Aging Neurosci. 2015; 7: 52 10.3389/fnagi.2015.00052 25954194 PMC4404917

[21] Swanson D, Block R, Mousa SA. Omega-3 fatty acids EPA and DHA: health benefits throughout life. Adv Nutr. 2012; 3: 1–7. 10.3945/an.111.000893 22332096 PMC3262608

[22] Baugh SD, Pabba PK, Barbosa J, Coulter E, Desai U, Gay JP, et al Design, synthesis, and in vivo activity of novel inhibitors of delta-5 desaturase for the treatment of metabolic syndrome. Bioorg Med Chem Lett. 2015; 25: 3836–3839. 10.1016/j.bmcl.2015.07.066 26235947

[23] Powell DR, Gay JP, Smith M, Wilganowski N, Harris A, Holland A, et al Fatty acid desaturase 1 knockout mice are lean with improved glycemic control and decreased development of atheromatous plaque. Diabetes Metab Syndr Obes. 2016; 9: 185–199. 10.2147/DMSO.S106653 27382320 PMC4922822

[24] Suzuki H, Fujimoto T, Yamamoto T. Patent WO2010087467 A1.

[25] Ntambi JM, Miyazaki M, Stoehr JP, Lan H, Kendziorski CM, Yandell BS, et al Loss of stearoyl-CoA desaturase-1 function protects mice against adiposity. Proc Natl Acad Sci U S A. 2002; 99: 11482–11486. 10.1073/pnas.132384699 12177411 PMC123282

[26] Miyahisa I, Suzuki H, Mizukami A, Tanaka Y, Ono M, Hixon MS, et al T-3364366 Targets the Desaturase Domain of Delta-5 Desaturase with Nanomolar Potency and a Multihour Residence Time. ACS Med Chem Lett. 2016; 7: 868–872. 10.1021/acsmedchemlett.6b00241 27660693 PMC5018866

[27] Bligh EG, Dyer WJ. A rapid method for total lipid extraction and purification. Patent WO2008089310A2. 1959; Can.J.Biochem.Physiol.: 911–917. 10.1139/o59-099 13671378

[28] Matthews DR, Hosker JP, Rudenski AS, Naylor BA, Treacher DF, Turner RC. Homeostasis model assessment: insulin resistance and beta-cell function from fasting plasma glucose and insulin concentrations in man. Diabetologia. 1985; 28: 412–419. 3899825 10.1007/BF00280883

[29] Tschop MH, Speakman JR, Arch JR, Auwerx J, Bruning JC, Chan L, et al A guide to analysis of mouse energy metabolism. Nat Methods. 2012; 9: 57–63. 10.1038/nmeth.1806 PMC3654855 22205519

[30] Speakman JR. Measuring energy metabolism in the mouse—theoretical, practical, and analytical considerations. Front Physiol. 2013; 4: 34 10.3389/fphys.2013.00034 23504620 PMC3596737

[31] Vessby B, Gustafsson IB, Tengblad S, Boberg M, Andersson A. Desaturation and elongation of Fatty acids and insulin action. Ann N Y Acad Sci. 2002; 967: 183–195. 12079847 10.1111/j.1749-6632.2002.tb04275.x

[32] Steffen LM, Vessby B, Jacobs DR Jr., Steinberger J, Moran A, Hong CP, et al Serum phospholipid and cholesteryl ester fatty acids and estimated desaturase activities are related to overweight and cardiovascular risk factors in adolescents. Int J Obes (Lond). 2008; 32: 1297–1304. 18560369 10.1038/ijo.2008.89 PMC2832613

[33] Warensjo E, Rosell M, Hellenius ML, Vessby B, De Faire U, Riserus U. Associations between estimated fatty acid desaturase activities in serum lipids and adipose tissue in humans: links to obesity and insulin resistance. Lipids Health Dis. 2009; 8: 37 10.1186/1476-511X-8-37 19712485 PMC2746208

[34] Saito E, Okada T, Abe Y, Odaka M, Kuromori Y, Iwata F, et al Abdominal adiposity is associated with fatty acid desaturase activity in boys: implications for C-reactive protein and insulin resistance. Prostaglandins Leukot Essent Fatty Acids. 2013; 88: 307–311. 10.1016/j.plefa.2013.01.005 23419767

[35] Park H, Hasegawa G, Shima T, Fukui M, Nakamura N, Yamaguchi K, et al The fatty acid composition of plasma cholesteryl esters and estimated desaturase activities in patients with nonalcoholic fatty liver disease and the effect of long-term ezetimibe therapy on these levels. Clin Chim Acta. 2010; 411: 1735–1740. 10.1016/j.cca.2010.07.012 20654606

[36] Kroger J, Zietemann V, Enzenbach C, Weikert C, Jansen EH, Doring F, et al Erythrocyte membrane phospholipid fatty acids, desaturase activity, and dietary fatty acids in relation to risk of type 2 diabetes in the European Prospective Investigation into Cancer and Nutrition (EPIC)-Potsdam Study. Am J Clin Nutr. 2011; 93: 127–142. 10.3945/ajcn.110.005447 20980488

[37] Stoffel W, Hammels I, Jenke B, Binczek E, Schmidt-Soltau I, Brodesser S, et al Obesity resistance and deregulation of lipogenesis in Delta6-fatty acid desaturase (FADS2) deficiency. EMBO Rep. 2014; 15: 110–120. 10.1002/embr.201338041 24378641 PMC4303455

[38] Sartipy P, Loskutoff DJ. Monocyte chemoattractant protein 1 in obesity and insulin resistance. Proc Natl Acad Sci U S A. 2003; 100: 7265–7270. 10.1073/pnas.1133870100 12756299 PMC165864

[39] Kanda H, Tateya S, Tamori Y, Kotani K, Hiasa K, Kitazawa R, et al MCP-1 contributes to macrophage infiltration into adipose tissue, insulin resistance, and hepatic steatosis in obesity. J Clin Invest. 2006; 116: 1494–1505. 10.1172/JCI26498 16691291 PMC1459069

[40] Rimoldi OJ, Finarelli GS, Brenner RR. Effects of diabetes and insulin on hepatic delta6 desaturase gene expression. Biochem Biophys Res Commun. 2001; 283: 323–326. 10.1006/bbrc.2001.4785 11327701

[41] Zhang Y, Proenca R, Maffei M, Barone M, Leopold L, Friedman JM. Positional cloning of the mouse obese gene and its human homologue. Nature. 1994; 372: 425–432. 10.1038/372425a0 7984236

[42] Hondares E, Rosell M, Diaz-Delfin J, Olmos Y, Monsalve M, Iglesias R, et al Peroxisome proliferator-activated receptor alpha (PPARalpha) induces PPARgamma coactivator 1alpha (PGC-1alpha) gene expression and contributes to thermogenic activation of brown fat: involvement of PRDM16. J Biol Chem. 2011; 286: 43112–43122. 10.1074/jbc.M111.252775 22033933 PMC3234861

[43] Wang MY, Lee Y, Unger RH. Novel form of lipolysis induced by leptin. J Biol Chem. 1999; 274: 17541–17544. 10364187 10.1074/jbc.274.25.17541

[44] Pisani DF, Ghandour RA, Beranger GE, Le Faouder P, Chambard JC, Giroud M, et al The omega6-fatty acid, arachidonic acid, regulates the conversion of white to brite adipocyte through a prostaglandin/calcium mediated pathway. Mol Metab. 2014; 3: 834–847. 10.1016/j.molmet.2014.09.003 25506549 PMC4264041

[45] Zhang LN, Gamo Y, Sinclair R, Mitchell SE, Morgan DG, Clapham JC, et al Effects of chronic oral rimonabant administration on energy budgets of diet-induced obese C57BL/6 mice. Obesity (Silver Spring). 2012; 20: 954–962. 22173576 10.1038/oby.2011.357

[46] Verty AN, Allen AM, Oldfield BJ. The effects of rimonabant on brown adipose tissue in rat: implications for energy expenditure. Obesity (Silver Spring). 2009; 17: 254–261. 19057531 10.1038/oby.2008.509

[47] Perwitz N, Wenzel J, Wagner I, Buning J, Drenckhan M, Zarse K, et al Cannabinoid type 1 receptor blockade induces transdifferentiation towards a brown fat phenotype in white adipocytes. Diabetes Obes Metab. 2010; 12: 158–166. 10.1111/j.1463-1326.2009.01133.x 19895638

