# Supplementary material for: A Novel Selective Inhibitor of Delta-5 Desaturase Lowers Insulin Resistance and Reduces Body Weight in Diet-Induced Obese C57BL/6J Mice
Source: PLoS One. 2016 Nov 10;11(11):e0166198. doi: 10.1371/journal.pone.0166198 (PMC5104425; doi:10.1371/journal.pone.0166198)
Supplement: S1 Fig — (DOCX) [file pone.0166198.s001.docx]

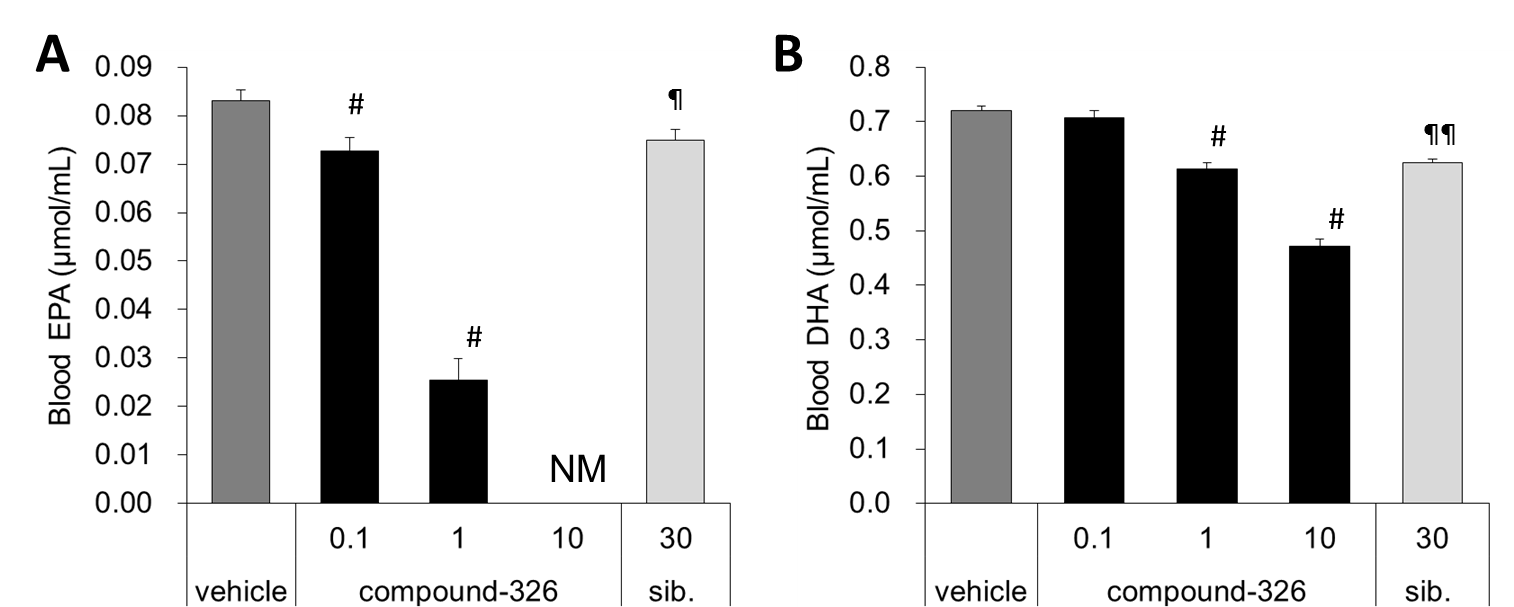


**S1 Fig. Effects of chronic treatment with compound-326 on blood EPA and DHA levels in DIO mice.**

DIO mice (initial BW; 31.1±1.6 g) were treated with compound-326 (0.1, 1, and 10 mg/kg), 30 mg/kg sibutramine, or vehicle p.o. for 6 weeks. (**A**) Changes in blood EPA concentrations after 6-week treatment. (**B**) Changes in blood DHA concentrations after 6-week treatment. NM stands for note measureable. Data are expressed as mean ± *SE*. (n=7). #*p*≤ 0.025 vs. DIO vehicle by Williams' test. ¶*p*≤ 0.05, ¶¶*p*≤ 0.01 vs. DIO vehicle by Student's t-test.
