# Supplementary material for: A Novel Selective Inhibitor of Delta-5 Desaturase Lowers Insulin Resistance and Reduces Body Weight in Diet-Induced Obese C57BL/6J Mice
Source: PLoS One. 2016 Nov 10;11(11):e0166198. doi: 10.1371/journal.pone.0166198 (PMC5104425; doi:10.1371/journal.pone.0166198)
Supplement: S2 Fig — (DOCX) [file pone.0166198.s002.docx]

#
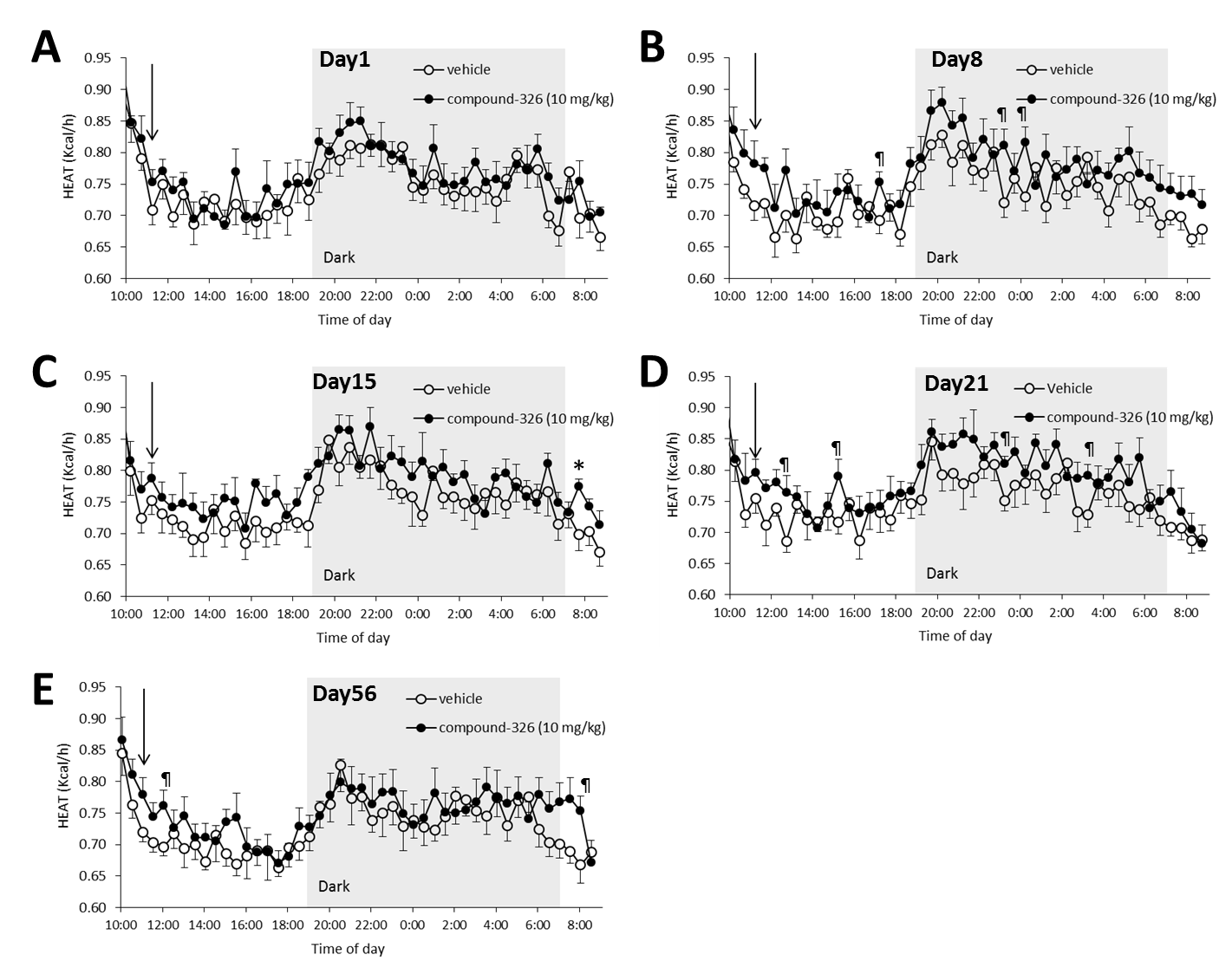


**S2 Fig. Effect of chronic treatment with compound-326 on energy expenditure in DIO mice.**

Energy expenditure (EE) was monitored on days 1 (**A**), 8 (**B**), 15 (**C**), 21 (**D**), and 56 (**E**) during chronic dosing study indicated in Fig 5. Arrows indicate the timing of drug administration. Data are expressed as mean ± *SE* (n=7). **p*≤ 0.05 vs. DIO vehicle by Aspin-Welch test. ¶*p*≤ 0.05 vs. DIO vehicle by Student's t-test.
