# Supplementary material for: A Novel Selective Inhibitor of Delta-5 Desaturase Lowers Insulin Resistance and Reduces Body Weight in Diet-Induced Obese C57BL/6J Mice
Source: PLoS One. 2016 Nov 10;11(11):e0166198. doi: 10.1371/journal.pone.0166198 (PMC5104425; doi:10.1371/journal.pone.0166198)
Supplement: S3 Fig — (DOCX) [file pone.0166198.s003.docx]

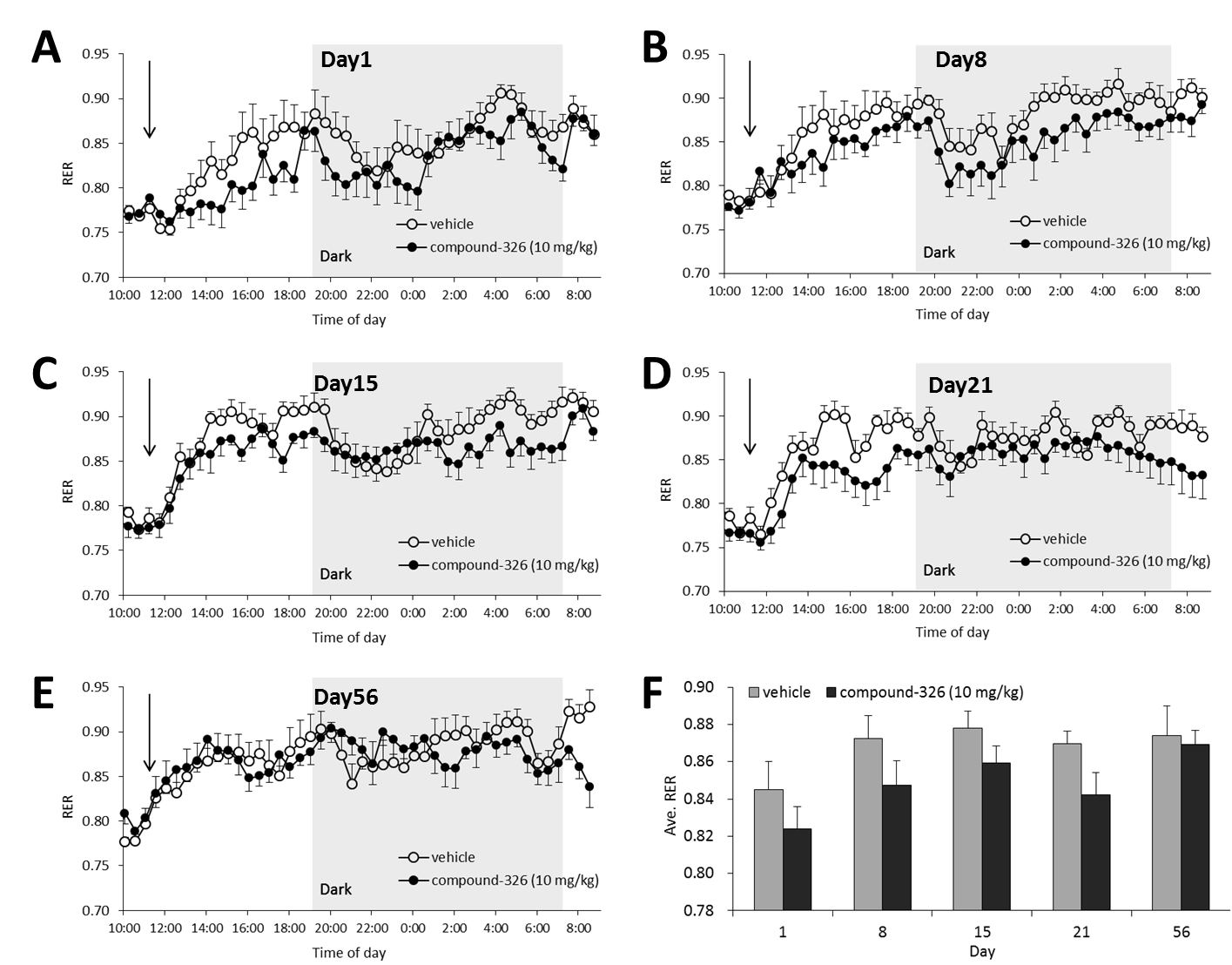


**S3 Fig. Effect of chronic treatment with compound-326 on respiratory exchange ratio in DIO mice.**

Respiratory exchange ratio (RER) was monitored on days 1 (**A**), 8 (**B**), 15 (**C**), 21 (**D**), and 56 (**E**) during chronic dosing study indicated in Fig 5. (**F**) Changes in averaged RER. Arrows indicate the timing of drug administration. Data are expressed as mean ± *SE* (n=7).
