# Supplementary material for: A Novel Selective Inhibitor of Delta-5 Desaturase Lowers Insulin Resistance and Reduces Body Weight in Diet-Induced Obese C57BL/6J Mice
Source: PLoS One. 2016 Nov 10;11(11):e0166198. doi: 10.1371/journal.pone.0166198 (PMC5104425; doi:10.1371/journal.pone.0166198)
Supplement: S4 Fig — (DOCX) [file pone.0166198.s004.docx]

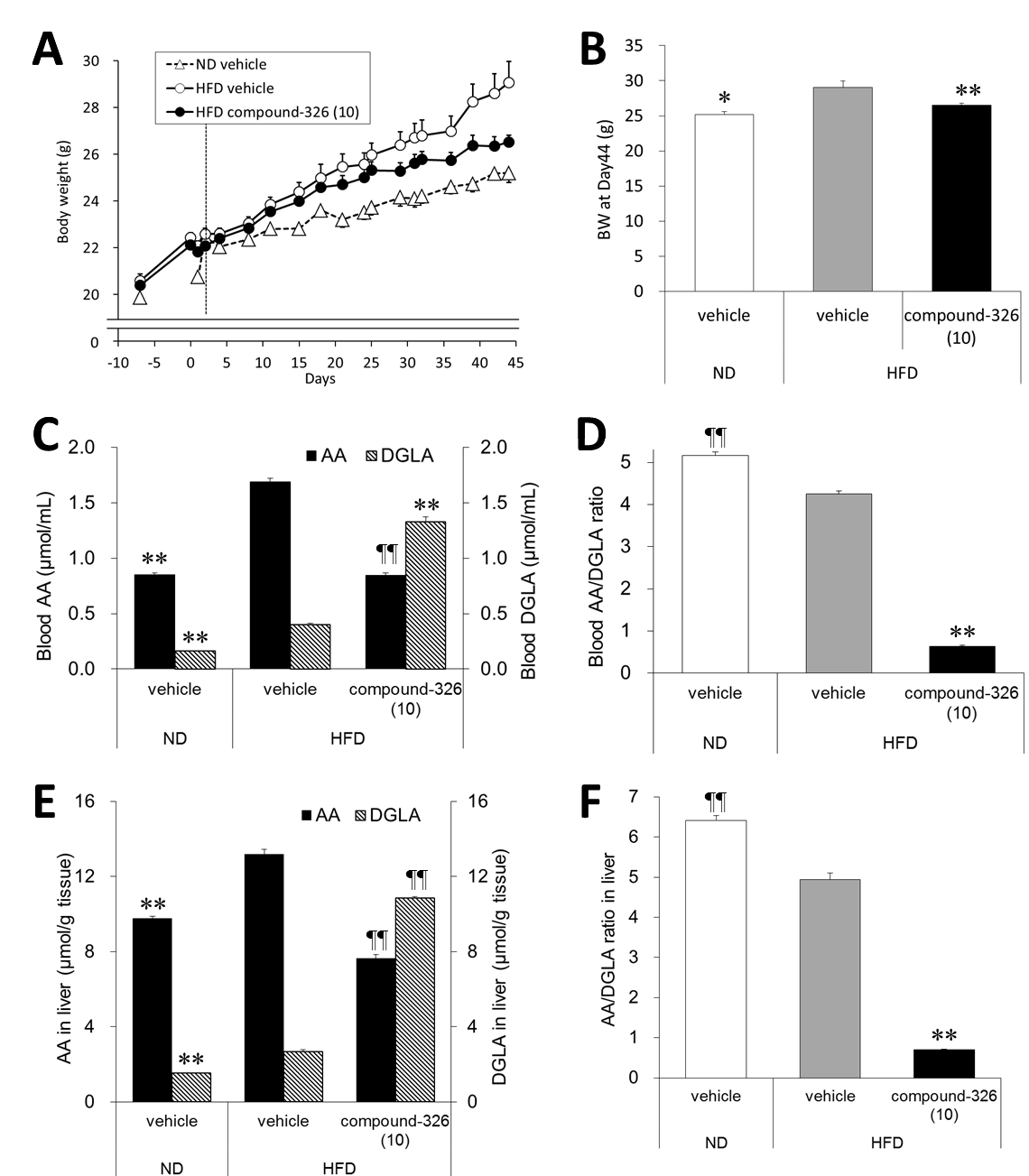


**S4 Fig. Effects of compound-326 on BW and AA and DGLA levels within the blood as well as liver in DIO mice.**

Chronic administration of compound-326 (10 mg/kg) significantly decreased BW in DIO mice. In these mice, increase in DGLA and decrease in AA in the blood as well as liver were observed after 86-week treatment with compound-326. (**A**) BW changes during the study. (**B**) BW at Day44. (**C**) Levels of AA and DGLA in the blood. (**D**) AA to DGLA ratio in the blood. (**E**) Levels of AA and DGLA in the liver. (**F**) AA to DGLA ratio in the liver. Data are expressed as mean ± *SE* (n=8-9). **p*≤ 0.05, ***p*≤ 0.01 vs. DIO vehicle by Aspin-Welch test. ¶¶*p*≤ 0.01 vs. DIO vehicle by Student's t-test.
