# Supplementary material for: A Novel Selective Inhibitor of Delta-5 Desaturase Lowers Insulin Resistance and Reduces Body Weight in Diet-Induced Obese C57BL/6J Mice
Source: PLoS One. 2016 Nov 10;11(11):e0166198. doi: 10.1371/journal.pone.0166198 (PMC5104425; doi:10.1371/journal.pone.0166198)
Supplement: S5 Fig — (DOCX) [file pone.0166198.s005.docx]

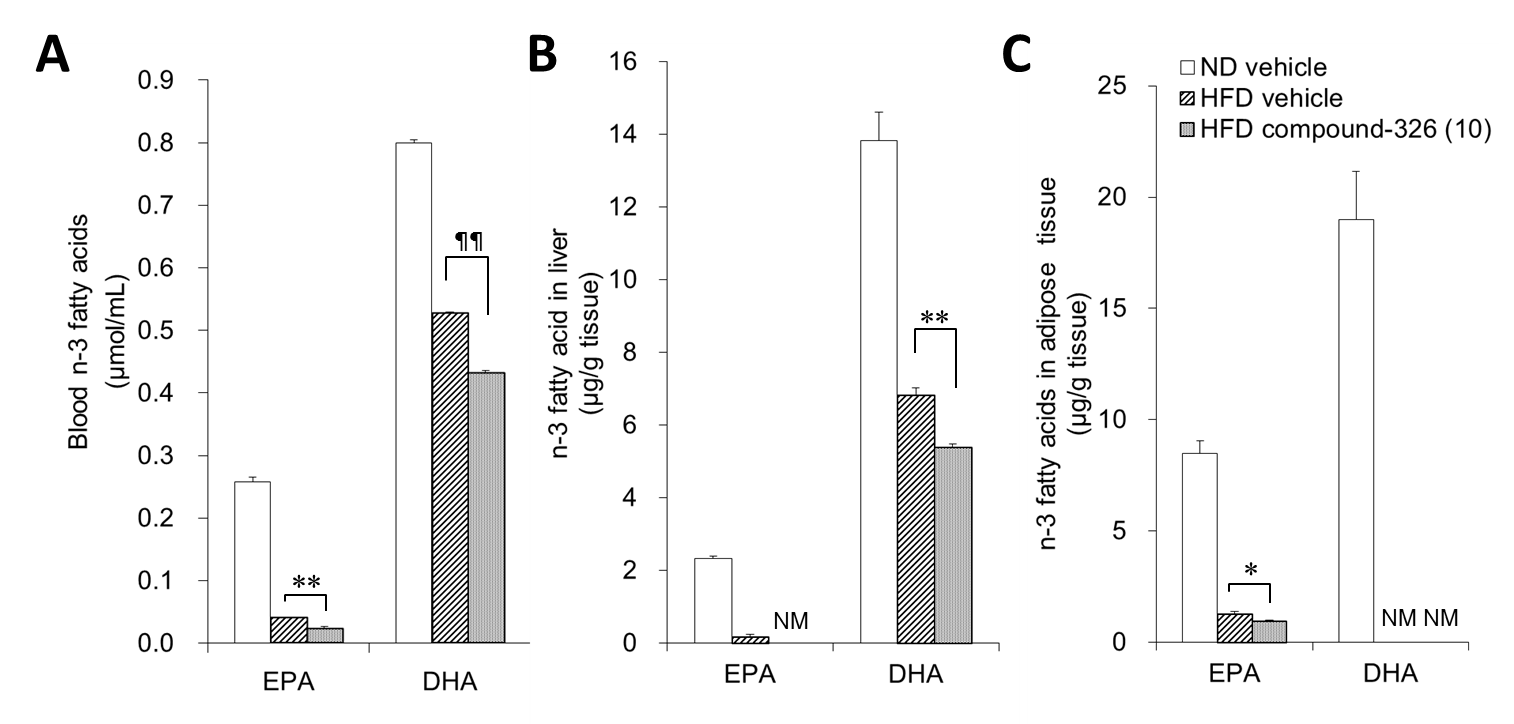


**S5 Fig. Effects of chronic treatment with compound-326 on blood and tissue EPA and DHA levels in HFD-fed mice.**

HFD-fed mice were treated with compound-326 (10 mg/kg), p.o. for 6 weeks. Mice fed a normal diet (ND) were used as normal reference. (**A**) Levels of EPA and DHA in the blood. (**B**) Levels of EPA and DHA in epididymal adipose tissue. (**C**) Levels of EPA and DHA in epididymal adipose tissue. NM stands for note measureable. Data are expressed as mean ± *SE* (n=8-9). **p*≤ 0.05 and ***p*≤ 0.01 vs. DIO vehicle by Aspin-Welch test. ¶¶*p*≤ 0.01 vs. DIO vehicle by Student's t-test.
