# Supplementary material for: A Novel Selective Inhibitor of Delta-5 Desaturase Lowers Insulin Resistance and Reduces Body Weight in Diet-Induced Obese C57BL/6J Mice
Source: PLoS One. 2016 Nov 10;11(11):e0166198. doi: 10.1371/journal.pone.0166198 (PMC5104425; doi:10.1371/journal.pone.0166198)
Supplement: S6 Fig — (DOCX) [file pone.0166198.s006.docx]

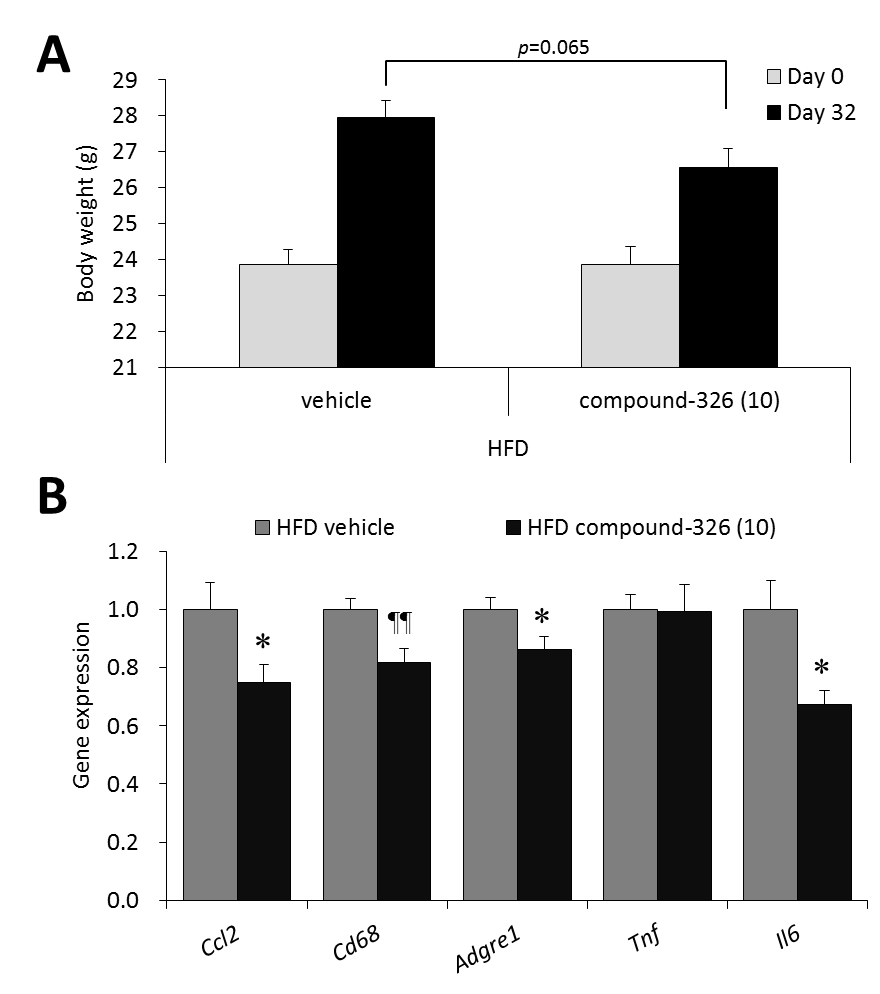


**S6 Fig. Effects of compound-326 on WAT gene expression in satellite DIO mice (Day32).**

In 6 weeks administration study of compound-326 (10 mg/kg), satellite animals were prepared to evaluate the gene expression levels in epididymal fat at the intermediate point (day 32). Mice fed a HFD (D12079B; Research diets, Inc.) from 5 weeks of age were divided into 2 groups at the age of 8 weeks (11 animals per group), and were administered 10 mg/kg compound-326 or vehicle for 32 days. After the treatment period, mice were sacrificed and epididymal fat were harvested for mRNA analysis. (**A**) Compound-326 administration showed a trend towards BW loss in DIO mice. (**B**) RT-qPCR was performed in epididymal fat tissue after 32 days treatment. Data are expressed as mean ± *SE* (n=11). **p*≤ 0.05 vs. DIO vehicle by Aspin-Welch test. ¶¶*p*≤ 0.01 vs. DIO vehicle by Student's t-test.
