# Supplementary material for: A Novel Selective Inhibitor of Delta-5 Desaturase Lowers Insulin Resistance and Reduces Body Weight in Diet-Induced Obese C57BL/6J Mice
Source: PLoS One. 2016 Nov 10;11(11):e0166198. doi: 10.1371/journal.pone.0166198 (PMC5104425; doi:10.1371/journal.pone.0166198)
Supplement: S1 Table — (DOCX) [file pone.0166198.s007.docx]

**S1 Table. Fatty acids composition of the high-fat diet (data are expressed as w/w%)**

| Fatty acids | | D12492 | D12079B |
| --- | --- | --- | --- |
| C2 | Acetic | 0 | 0 |
| C4 | Butyric | 0 | 0.64 |
| C6 | Caproic | 0 | 0.38 |
| C8 | Caprylic | 0 | 0.22 |
| C10 | Capric | 0.01 | 0.50 |
| C12 | Lauric | 0.03 | 0.56 |
| C14 | Myristic | 0.36 | 2.00 |
| C14:1 | Myristoleic | 0 | 0.30 |
| C15 |  | 0.03 | - |
| C16 | Palmitic | 6.45 | 5.34 |
| C16:1 | Palmitoleic | 0.44 | 0.46 |
| C16:2 |  | 0 | - |
| C16:3 |  | 0 | - |
| C16:4 |  | 0 | - |
| C17 |  | 0.12 | - |
| C17:1 |  | 0 | - |
| C18 | Stearic | 3.48 | 2.44 |
| C18:1 | Oleic | 11.19 | 5.26 |
| C18:2 | Linoleic | 9.45 | 1.06 |
| C18:3 | Linolenic | 0.67 | 0.29 |
| C18:4 | Stearidonic | 0 | 0 |
| C20 | Arachidic | 0.05 | 0.19 |
| C20:1 |  | 0.19 | 0 |
| C20:2 |  | 0.26 | - |
| C20:3 |  | 0.04 | - |
| C20:4 | Arachidonic | 0.09 | 0 |
| C20:5 | Eicosapentaenoic | 0 | 0 |
| C21:5 |  | 0 | - |
| C22, | Behenic | 0 | 0 |
| C22:1 | Erucic | 0 | 0 |
| C22:4 | Clupanodonic | 0 | 0 |
| C22:5 | Docosapentaenoic | 0.03 | 0 |
| C22:6 | Docosahexaenoic | 0 | 0 |
| C24 | Lignoceric | 0 | 0 |
| C24:1 |  | 0 | - |
|  |  |  | (%) |
